# Double Knockout of Peroxiredoxin 4 (Prdx4) and Superoxide Dismutase 1 (Sod1) in Mice Results in Severe Liver Failure

**DOI:** 10.1155/2018/2812904

**Published:** 2018-06-27

**Authors:** Takujiro Homma, Toshihiro Kurahashi, Jaeyong Lee, Atsunori Nabeshima, Sohsuke Yamada, Junichi Fujii

**Affiliations:** ^1^Department of Biochemistry and Molecular Biology, Graduate School of Medical Sciences, Yamagata University, Yamagata, Japan; ^2^Department of Pathology and Cell Biology, School of Medicine, University of Occupational and Environmental Health, Kitakyushu, Japan

## Abstract

Mice that are deficient in superoxide dismutase 1 (Sod1), an antioxidative enzyme, are susceptible to developing liver steatosis. Peroxiredoxin 4 (Prdx4) catalyzes disulfide bond formation in proteins via the action of hydrogen peroxide and hence decreases oxidative stress and supports oxidative protein folding for the secretion of lipoproteins. Because elevated reactive oxygen species induce endoplasmic reticulum stress, this negative chain reaction is likely involved in the development of nonalcoholic fatty liver diseases and more advanced steatohepatitis (NASH). In the current study, we generated Prdx4 and Sod1 double knockout (DKO; Prdx4^−/y^Sod1^−/−^) mice and examined whether the combined deletion of Prdx4 and Sod1 aggravated liver pathology compared to single knockout and wild-type mice. The secretion of triglyceride-rich lipoprotein was strikingly impaired in the DKO mice, leading to aggravated liver steatosis. Simultaneously, the activation of caspase-3 in the liver was observed. The hyperoxidation of Prdxs, a hallmark of oxidative stress, occurred in different isoforms that are uniquely associated with Sod1^−/−^ and Prdx4^−/y^ mice, and the effect was additive in DKO mouse livers. Because DKO mice spontaneously develop severe liver failure at a relatively young stage, they have the potential for use as a model for hepatic disorders and for testing other potential treatments.

## 1. Introduction

Nonalcoholic fatty liver disease (NAFLD), defined as the accumulation of excessive levels of fat in the livers of subjects without a history of alcohol overconsumption, is a common chronic liver disease [1]. When NAFLD patients suffer from a further deteriorating insult, such as oxidative stress and/or endoplasmic reticulum (ER) stress, their condition advances to nonalcoholic steatohepatitis (NASH) [2].

Included among the deteriorating factors is oxidative stress, which occurs in various situations, such as insufficient levels of antioxidants, an elevated production of reactive oxygen species (ROS) by inflammation, and an excessive intake of drugs. Superoxide dismutase (Sod) scavenges superoxide radicals, a primary ROS produced in the body, and hence plays a central role in protection against oxidative stress [3]. Of the three genes that encode Sod in mammals, Sod1 is present largely in the cytoplasm and partly in the intermembrane space of mitochondria [4]. Among the multiple phenotypes, Sod1-knockout (Sod1^−/−^) mice show a shortened life span with an increased incidence of liver cancer [5–7] and abnormal lipid metabolism [8, 9]. Whereas visceral fat levels in Sod1^−/−^ mice are lower than that of WT mice [10], lipids accumulate at increased levels in the livers of Sod1^−/−^ mice [8, 9]. The impaired secretion of very low density lipoproteins (VLDL), which are rich in triglycerides (TG) and contain apolipoprotein B (apoB) [9], from the liver would at least partly explain the lipid accumulation in the livers of Sod1^−/−^ mice. In fact, it has been reported that superoxide plays a role in the degradation of apoB via stimulating the lipid peroxidation of polyunsaturated fatty acids [11]. We previously reported that oxidative stress is elevated in primary Sod1^−/−^ hepatocytes, resulting in the activation of lipogenesis via the activation of SREBP1c and lipid droplet accumulation [12]. This abnormal lipid metabolism appears to be associated with ER stress, which triggers the stimulation of lipogenesis but suppresses lipoprotein secretion.

Peroxiredoxin 4 (Prdx4), a member of the Prdx family, plays an antioxidative role by reducing hydrogen peroxide levels and suppressing the signaling role of hydrogen peroxide in mammals [13]. Unlike the other members, Prdx4 is predominantly localized to the ER and partly secreted to the extracellular space [14–16]. Prdx4 mediates the sulfoxidation of members of the protein disulfide isomerase (PDI) family, which consequently introduces disulfide bonds in newly synthesized proteins, which is accompanied by the elimination of hydrogen peroxide, thereby suppressing oxidative stress in the ER [16]. Whereas Prdx4-knockout (Prdx4^−/y^) mice show no apparent phenotypic abnormality except for testicular atrophy [17], it has been reported that the human Prdx4 transgene renders nongenetic models of NAFLD and/or type 2 diabetes mellitus resistant to the progression of the pathology [18]. Thus, it is assumed that Prdx4 has a protective role against hepatic disorders by reducing oxidative stress and synergistically suppressing liver steatosis and inflammation in livers.

Both oxidative stress and ER stress have been reported to induce the accumulation of lipid droplets in vitro [12, 19, 20]. ROS accelerate disulfide bridge formation between nearby sulfhydryl groups in a nonspecific manner, which increases the rate of occurrence of misfolded proteins in the ER, thereby leading to ER stress [21]. Because oxidative stress is a potential cause of this type of ER stress, we hypothesized that ER stress elicited by a Prdx4 deficiency may exacerbate oxidative stress induced-liver pathology in Sod1^−/−^ mice.

In the current study, we generated Prdx4 and Sod1 double-knockout (DKO; Prdx4^−/y^Sod1^−/−^) mice and investigated whether the combined deletion of Prdx4 and Sod1 aggravated liver pathology compared to mice in which only one of these genes had been knocked out and wild-type mice. The results revealed that the DKO mice spontaneously develop severe liver failure accompanied by increased levels of oxidative stress and the ER stress occurs early in life, suggesting that Sod1 and Prdx4 have protective roles against liver failure.

## 2. Materials and Methods

### 2.1. Mice

C57BL/6N (wild-type, WT) mice were purchased from Japan SLC (Hamamatsu, Japan). Sod1^−/−^ mice, originally established by Matzuk et al. [22], were purchased from the Jackson Laboratories (Bar Harbor, ME, USA) and had been backcrossed to a C57BL/6N background. Prdx4^−/y^ mice with a C57BL/6N background were generated using the gene-targeting technique in our institute [17]. Because female Sod1^−/−^ mice are not very fertile [22, 23], Prdx4^−/y^Sod1^+/−^ females were bred with Prdx4^−/y^Sod1^−/−^ (DKO (double knockout)) males. DKO mice were born at the predicted Mendelian ratio. Genotypic analyses of the mice were performed using PCR with specific primers. Adult (8–12-week-old) WT, Sod1^−/−^, Prdx4^−/y^, and DKO male mice were used in this study unless otherwise stated. All of the mice were weaned at 30 days of age and fed a standard diet (PicoLab 5053, LabDiet, St. Louis, MO, USA) ad libitum with free access to water. The animal room was maintained under specific pathogen-free conditions at a constant temperature of 20–22°C with a 12 h alternating light-dark cycle. Animal experiments were performed in accordance with the Declaration of Helsinki under protocols approved by the Animal Research Committee at Yamagata University.

### 2.2. Blood Tests

Blood was collected from the tail vein or the heart at the time of liver harvest in the presence of an excess of ethylenediaminetetraacetic acid. After centrifugation at 800 ×g for 5 min, the levels of plasma alanine transaminase (ALT) and aspartate transaminase (AST) were determined using FUJI DRI-CHEM 3500 V and FUJI DRI-CHEM slides (Fuji film). Plasma triglycerides (TG) and nonesterified fatty acids (NEFA) were measured by LabAssay™ Triglyceride and LabAssay NEFA (Wako Pure Chemical Industries, Osaka, Japan), respectively.

### 2.3. Histological Analyses of Liver

Livers dissected from the mice were fixed in 15% buffered formalin followed by embedding in paraffin. Sections (3–5 *μ*m) were stained with hematoxylin and eosin (H&E). To visualize hepatic lipid accumulation, the frozen liver sections were stained with an Oil Red O solution, as described previously [24].

### 2.4. Assay for TG Secretion

After a 4 h fast, mice were injected intraperitoneally with tyloxapol (1 g/kg; Sigma-Aldrich Corporation, St. Louis, MO, USA). Blood samples were withdrawn at 0 and 180 min after the injection, and plasma TG concentrations were determined as described above.

### 2.5. Intragastric Fat Load

After a 12 h fast, mice were administered 300 *μ*l of olive oil by gavage. Blood samples were drawn at 0, 1, 2, and 3 h after the administration, and plasma TG concentrations were determined as described above.

### 2.6. Isolation of Hepatocytes and Primary Culture

Mice at 8–10 weeks of age were used for preparing hepatocytes. Livers of anaesthetized mice were perfused with Ca^2+^-free Krebs ringer-HEPES buffer, pH 7.2, containing collagenase (Wako) and a trypsin inhibitor (Sigma-Aldrich Corporation) at 37°C. The resulting cells (1.5 × 10^6^ cells/6 cm dish) were transferred to collagen-coated plastic dishes and cultivated in William's medium (Sigma-Aldrich Corporation) supplemented with 10% fetal bovine serum, 100 units penicillin, 0.1 mg/ml streptomycin, 1 mM GlutaMAX supplement (Thermo Fisher Scientific, Waltham, MA, USA), and nonessential amino acids under an atmosphere of CO_2_ at 37°C.

### 2.7. Detection of Intracellular Lipids by Nile Red Staining

Cultured hepatocytes were rinsed with phosphate-buffered saline (PBS) and fixed in 4% formaldehyde solution for 1 h. After 2 washings with PBS, the cells were stained with 0.4 mM Nile Red and 1 *μ*g/ml DAPI for 15 min in the dark. For double staining, cells were permeabilized with 0.5% Tween-20 in PBS for 10 min at room temperature, blocked for 1 h at room temperature in TBST containing 5% skim milk, and incubated overnight at 4°C with an anti-PLIN2 antibody (Progen, GP40, 1 : 100) in TBST containing 1% skim milk. After three washes in PBS, the cells were incubated with the Alexa Fluor®-conjugated goat anti-guinea pig antibody (1 : 200) for 90 min at room temperature. All images were obtained using a confocal laser-scanning microscope (Zeiss LSM 700, Germany).

### 2.8. Protein Preparation

Livers were weighed and homogenized in 5 vol. of RIPA buffer, which contains 50 mM Tris-HCl, pH 8.0, 150 mM NaCl, 1% Nonidet P-40, 0.5% deoxycholate, and 0.1% SDS, supplemented with a protease inhibitor cocktail (Sigma-Aldrich Corporation). After centrifugation at 15,000 rpm, the protein concentration in the supernatant was determined using a BCA protein assay reagent (Thermo Fisher Scientific).

### 2.9. Western Blotting

Aliquots of protein (20–30 *μ*g) were separated by SDS-polyacrylamide gel electrophoresis (SDS-PAGE) and blotted onto polyvinylidene difluoride (PVDF) membranes (EMD Millipore, Temecula, CA, USA). The blots were blocked with 5% skim milk in Tris-buffered saline containing 0.1% Tween-20 and were then incubated with the antibodies. The primary antibodies used were anti-Prdx-SO_2/3_ (ab16830; Abcam, Cambridge, UK), anti-SOD1 [25], anti-Prdx1 [25], anti-Prdx2 (LF-PA0091; AbFrontier, Seoul, Korea), anti-Prdx3 (LF-MA0044; AbFrontier), anti-Prdx4 [17], anti-endoplasmic reticulum stress-C/EBP homologous protein (CHOP) (sc-7351; Santa Cruz Biotechnology, CA, USA), anti-PDI (sc-20132; Santa Cruz Biotechnology), anti-IRE1*α* (number 3294; Cell Signaling Technology, Danvers, MA, USA), anticleaved caspase-3 (number 9664; Cell Signaling Technology), and anti *β*-actin (sc-69879; Santa Cruz Biotechnology). Horseradish peroxidase-conjugated goat anti-rabbit, anti-mouse, and anti-guinea pig IgG antibodies (Santa Cruz Biotechnology) were used as the secondary antibodies. After washing, immune reactive bands were detected by measuring the chemiluminescence using Immobilon Western chemiluminescent horseradish peroxidase substrate (EMD Millipore, Temecula, CA, USA) on an image analyzer (ImageQuant LAS 500; GE Healthcare Life Sciences, Buckinghamshire, UK).

### 2.10. Measurement of Total Glutathione

Total glutathione was determined using a glutathione reductase and NADPH-coupled reaction with 5,5′-dithiobis(2-nitrobenzoic acid), as previously described [26]. Briefly, livers were weighed and homogenized in a 5% metaphosphoric acid/0.6% sulfosalicylic acid solution. After centrifugation at 3000 ×g for 10 min at 4°C, the supernatant was used for the assay.

### 2.11. Statistical Analysis

Statistical analyses were performed using the Student *t*-test and one-way or two-way ANOVA, followed by the Tukey-Kramer test for multiple groups. A *P* value of less than 0.05 was considered to be significant.

## 3. Results

### 3.1. DKO Mice Develop Severe Liver Damage

**Figure 1:**
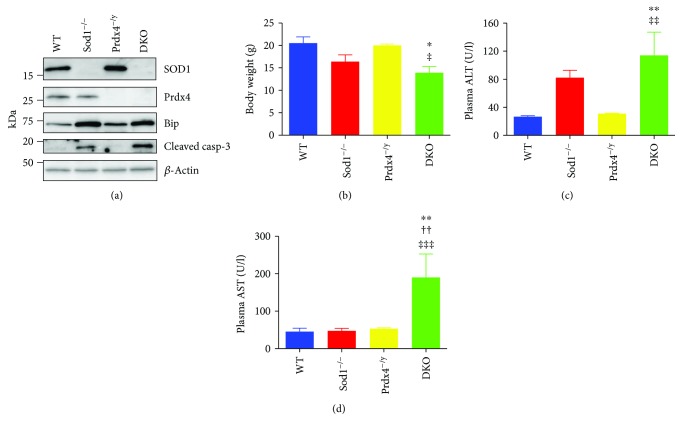
Body weight, plasma ALT, and AST activities in mice. (a) Representative plots of SOD1, Prdx4, Bip, cleaved caspase-3 (cleaved casp-3), and *β*-actin in whole liver lysates of mice with the indicated genotypes. (b) Body weight of mice with the indicated genotypes. Data are expressed as the mean ± SEM. Number of mice: WT: *n* = 3, Sod1^−/−^: *n* = 3, Prdx4^−/y^: *n* = 4, and DKO: *n* = 5. ^∗^ *P* < 0.05 versus WT mice. ^‡^ *P* < 0.05 versus Prdx4^−/y^ mice. (c) Plasma ALT activities in mice with the indicated genotypes. Data are expressed as the mean ± SEM. Number of mice: WT: *n* = 5, Sod1^−/−^: *n* = 6, Prdx4^−/y^: *n* = 10, and DKO: *n* = 6. ^∗∗^ *P* < 0.01 versus WT mice. ^‡‡^ *P* < 0.01 versus Prdx4^−/y^ mice. (d) Plasma AST activities in mice with the indicated genotypes. Data are expressed as the mean ± SEM. Number of mice: WT: *n* = 5, Sod1^−/−^: *n* = 7, Prdx4^−/y^: *n* = 10, and DKO: *n* = 6. ^∗∗^ *P* < 0.01 versus WT mice. ^††^ *P* < 0.01 versus Sod1^−/−^ mice. ^‡‡‡^ *P* < 0.001 versus Prdx4^−/y^ mice.

To determine whether Prdx4 ablation affects liver pathology induced by a Sod1 deficiency, we generated Prdx4^−/y^Sod1^−/−^ (DKO (double-knockout)) mice by intercrossing each single-knockout (KO) mouse. We first confirmed that the DKO mice did not express either Prdx4 or Sod1 in the liver (Figure 1(a)). An ER chaperon Bip was upregulated in the livers of Prdx4^−/y^, Sod1^−/−^, and DKO mice compared to the WT mice, indicating the increased ER stress in these mice. Moreover, the cleaved form of caspase-3, a marker of apoptotic cell death, was observed in the livers of Sod1^−/−^ mice and more prominent in the DKO mice, indicating that spontaneous liver damage had occurred in the DKO mice.

We then measured body weights and plasma ALT and AST levels among the DKO mice, the singly knockout mice, and WT mice to determine whether any relationship existed between these barometers of health and genotypes. As observed in Figure 1(b), the DKO mice were lighter in weight (versus WT, and Prdx4^−/y^ mice). The body weights of the Sod1^−/−^ mice also tended to be lower than those of the WT mice, consistent with previous report [27]. Plasma ALT levels were elevated by about threefold in the DKO mice compared to the WT mice (Figure 1(c)), consistent with the results of Western blotting. Although not statistically significant, there was a tendency for the ALT levels to be higher in the DKO mice than in the Sod1^−/−^ mice. In line with the increased ALT levels, a marked increase in plasma AST levels was observed in the DKO mice compared with the other genotypic groups of mice (Figure 1(d)). Different from the elevation in ALT levels, however, AST levels in the plasma were unchanged in Sod1^−/−^ mice. Plasma levels of blood urea nitrogen (BUN) in the DKO mice tended to be higher than those of the WT and Prdx4^−/y^ mice and were comparable to those in Sod1^−/−^ mice, although they were within the acceptable range (Supplementary Figure 1). Collectively, these results suggest that liver damage in the DKO mice was the most severe among this genetic group and might be associated with the lower body weights that were observed.

**Figure 2:**
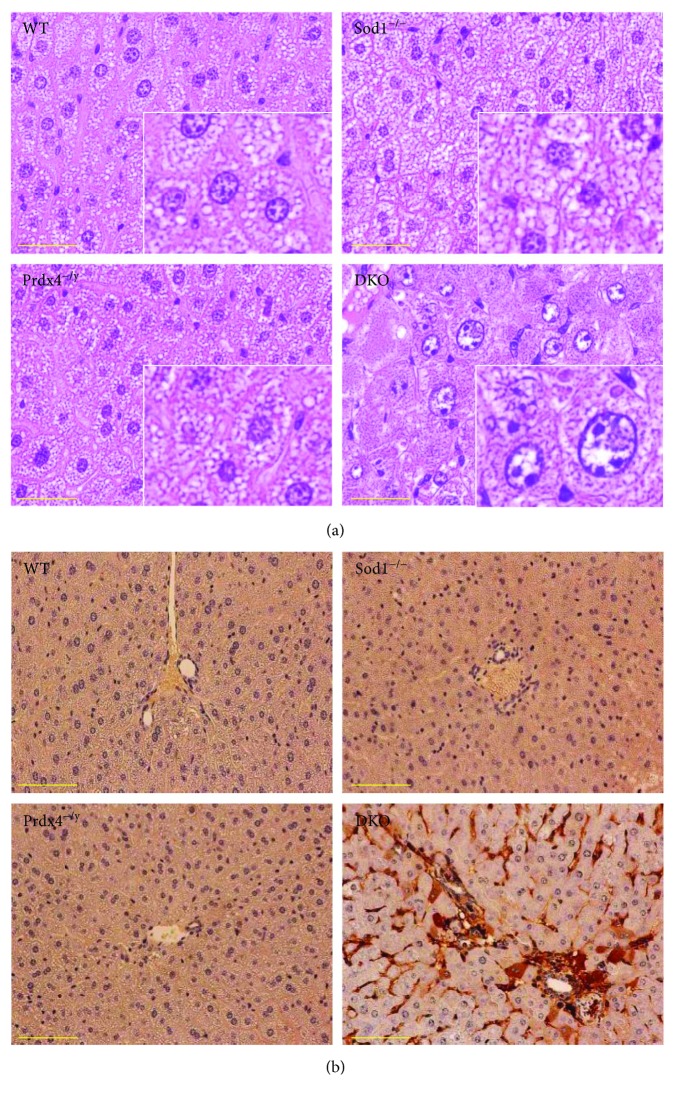
Histological analysis of the liver pathology in DKO mice. (a) Representative images of hematoxylin and eosin- (H&E) stained livers from the indicated genotypes. Inset: high-magnification images of corresponding liver sections. Note that there were degenerative changes, as evidenced by the ballooning of hepatocytes in livers of DKO mice. Scale bars: 50 *μ*m. (b) Immunohistochemical staining of cleaved caspase-3 in livers with the indicated genotypes. Nuclei were stained with hematoxylin. Scale bars: 50 *μ*m.

To characterize the liver pathology of DKO mice, we performed H&E staining of liver sections of all four genotypic mice (Figure 2(a)). The livers of Sod1^−/−^ mice showed a ballooning of hepatocytes, and the sizes of intracytoplasmic vacuolar structures were increased, which was characteristically similar to lipid droplets reported for Sod1^−/−^ mice [9], while no histological signs of pathology were observed in the livers of Prdx4^−/y^ mice compared to WT mice. In the case of DKO mice, severe degenerative changes in the hepatocytes were observed, as evidenced by the presence of cells with enlarged nuclei. To further confirm the existence of hepatic damage, we performed immunohistochemical staining for cleaved caspase-3 (Figure 2(b)). Consistent with the histological observations, the DKO mouse livers showed a pronounced staining for anticleaved caspase-3, indicating the presence of a large number of dying cells. However, there appeared to be a difference in the detection of cleaved caspase-3 between the immunoblot data in Figure 1(a) and immunohistochemical data in Figure 2(b). Although there was also a relatively strong protein band on Sod1^−/−^, no staining was detected in the immunohistochemical data. This slight discrepancy could be caused by a difference in detection sensitivity of the two methods because immunoblotting is a generally more sensitive method than immunohistochemistry. In contrast, the staining profiles of Sod1^−/−^ or Prdx4^−/y^ mouse hepatocytes were indistinguishable from those for WT mice. These observations indicate that a Prdx4 deficiency, together with coincidental oxidative stress caused by Sod1 ablation, was responsible for the observed spontaneous hepatic damage.

### 3.2. Reduced VLDL Production Is Associated with Hepatic Steatosis in DKO Mice

**Figure 3:**
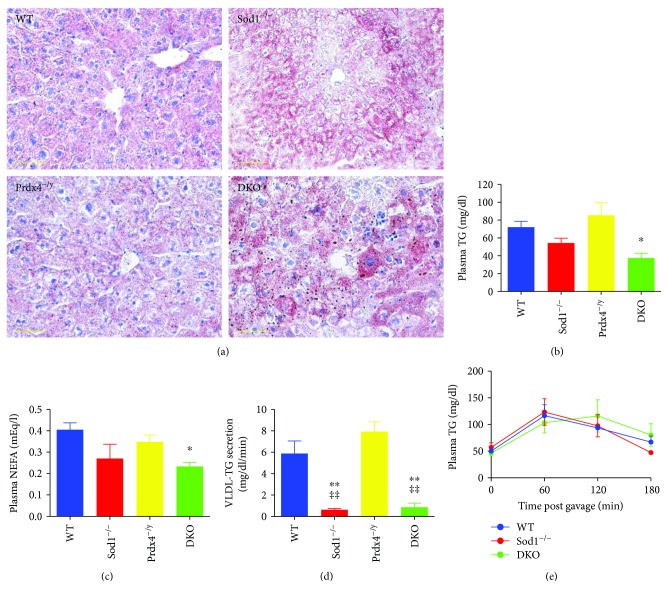
Lipid accumulation in livers of DKO mice. (a) Representative images of Oil Red O-stained livers with the indicated genotypes. Scale bars: 50 *μ*m. (b) Plasma TG levels in mice with the indicated genotypes. Data are expressed as the mean ± SEM. Number of mice: WT: *n* = 5, Sod1^−/−^: *n* = 7, Prdx4^−/y^: *n* = 10, and DKO: *n* = 6. ^∗^ *P* < 0.05 versus WT mice. (c) Plasma NEFA levels in mice with the indicated genotypes. Data are expressed as the mean ± SEM. Number of mice: WT: *n* = 5, Sod1^−/−^: *n* = 5, Prdx4^−/y^: *n* = 6, and DKO: *n* = 5. ^∗^ *P* < 0.05 versus WT mice. (d) VLDL-TG secretion in mice. Plasma TG levels were measured before and 180 min after intravenous tyloxapol injection. TG secretion rate during the 180 min experiment was calculated. Data are expressed as the mean ± SEM (*n* = 3–5). ^∗∗^ *P* < 0.01 versus WT mice. ^‡‡^ *P* < 0.01 versus Prdx4^−/y^ mice. (e) Plasma TG levels were measured at 60 min intervals after the oral administration of olive oil. Data are expressed as means ± SEM (*n* = 3–4). No difference was observed between the different genotypes.

We next performed Oil Red O staining of liver sections in these mice to characterize the intracytoplasmic vacuolar structures (Figure 3(a)). The results clearly showed the accumulation of larger numbers of lipid droplets in Sod1^−/−^ mouse livers than in WT mouse livers, consistent with previous reports [8, 9, 28], while no difference was found between Prdx4^−/y^ mouse livers and WT mouse livers. In the case of the DKO mouse livers, the numbers of lipid droplets were enhanced considerably compared to Sod1^−/−^ mouse livers.

The plasma levels of TG (Figure 3(b)) and NEFA (Figure 3(c)) were somewhat decreased in the DKO mice compared to the WT mice. While it is known that Sod1^−/−^ mice show abnormal lipid metabolism [9], these data suggest that the dysregulation of lipid metabolism was more severe in the DKO mice than in the Sod1^−/−^ mice. To determine if a decreased VLDL secretion was involved in hepatic steatosis in the DKO mice, we ip injected tyloxapol to inhibit intravascular TG hydrolysis and measured the entry of VLDL-TG into plasma [29]. The rates of VLDL-TG secretion were similarly quite slow in both Sod1^−/−^ and DKO mice (Figure 3(d)), suggesting a malfunction in the VLDL secretion process from the liver, consistent with the accumulation of TG in hepatocytes. We next compared the clearance of TG from the circulation in mice after an oral administration of olive oil. As shown in Figure 3(e), no difference in the TG clearance rate or the area under the curve (AUC) was found, indicating that the capacity for lipid consumption among the groups of mice was the same (WT: 16130 ± 728; Sod1^−/−^: 16390 ± 2641; and DKO: 16958 ± 3916 min·mg/dl). This result indicates that the consumption of chylomicron TG by tissues, such as adipose tissue, and their eventual uptake by the liver were not impaired in the DKO mice. Thus, these results indicate that the absence of Sod1 was a major factor in the reduced VLDL secretion from the liver but had no effect on TG consumption. The double ablation of SOD1 and Prdx4 appeared to impair VLDL secretion further than for the SOD1 single knockout.

### 3.3. In Vitro Experiments into the Molecular Mechanism for Hepatocyte Damage and Lipid Droplet Accumulation

**Figure 4:**
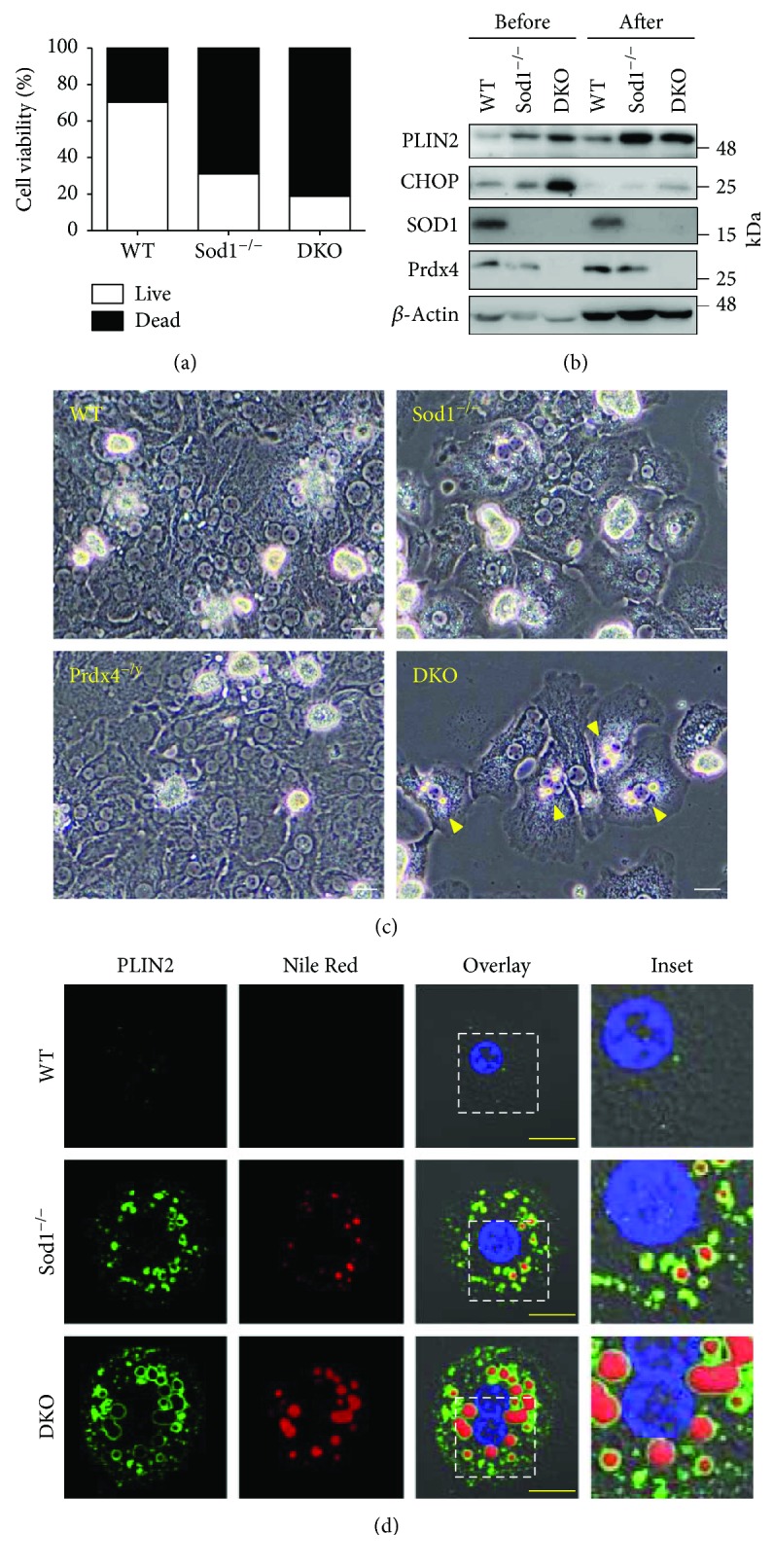
Oxidative stress induced lipid accumulation in DKO hepatocytes. (a) The average viability of isolated hepatocytes from WT, Sod1^−/−^, and DKO mice. Cell counting of either live (white columns) or dead cells (black columns), as measured by the Trypan blue exclusion test. (b) Representative blots of PLIN2, CHOP, SOD1, Prdx4, and *β*-actin in primary cultured hepatocytes. Proteins extracted from the hepatocytes before and 24 h after culturing were subjected to Western blotting. (c) The morphology of primary cultured hepatocytes of all four genotypes. The DKO hepatocytes containing large amounts of lipid droplets are indicated by yellow arrows. Scale bars: 20 *μ*m. (d) Representative images of DAPI- (blue), PLIN2- (green), and Nile Red- (red) stained primary cultured hepatocytes. Scale bars: 20 *μ*m.

In a preliminary experiment, we examined hepatocytes isolated from Prdx4^−/y^ mice and compared them to those from WT mice. Examination of some proteins that are involved in ER stress showed virtually no difference except for a trend showing an increase in PDI during the cell culture (Supplementary Figure 2). To gain insights into the molecular mechanisms underlying the development of liver pathology elicited in DKO mice, we isolated hepatocytes from DKO, Sod1^−/−^, and WT mice and subjected them to primary culture. Hepatocytes isolated from Sod1^−/−^ mice showed a lower viability than those from WT mice, and hepatocytes from DKO mice showed the lowest viability (Figure 4(a)), consistent with the hepatic damage observed *in vivo*. The level of PLIN2 expression was consistently higher at 24 h compared to the moment of isolation from livers (Figure 4(b)). The presence of the ER stress-regulated apoptosis inducer CHOP [30] was originally high in the DKO hepatocytes, but the levels were lower in all groups of cells at 24 h after the start of the culture.  Figure 4(c) shows the morphology of primary cultured hepatocytes for all four genotypes. While there was virtually no difference in the morphology of Prdx4^−/y^ from that of WT hepatocytes, the SOD1 deficiency caused massive damage in hepatocytes under cultured conditions. We then treated the primary cultured hepatocytes with Nile Red, a lipophilic fluorescent probe, and also immunostained for PLIN2, a lipid droplet coat protein produced by hepatocytes in humans and rodents [31]. While substantial numbers of lipid droplets accumulated in the Sod1^−/−^ hepatocytes, as we reported previously [12], a larger number of lipid droplets accumulated in the DKO hepatocytes compared with the Sod1^−/−^ hepatocytes (Figure 4(d)). Because the viability of DKO hepatocytes was extremely low, it is possible that cells with severe ER stress were dead and only modestly damaged cells had survived under cultural conditions.

### 3.4. Oxidative Stress and Antioxidative Systems in DKO Mice

**Figure 5:**
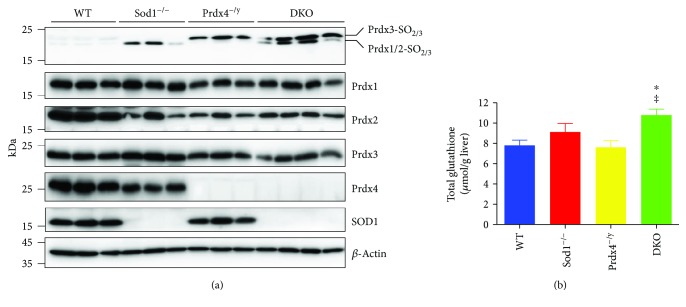
Levels of hyperoxidized Prdx and hepatic glutathione. (a) Western blots of Prdx-SO_2/3_, Prdx1, Prdx2, Prdx3, Prdx4, SOD1, and *β*-actin in whole liver lysates of mice with the indicated genotypes. Number of mice: WT: *n* = 3, Sod1^−/−^: *n* = 3, Prdx4^−/y^: *n* = 3, and DKO: *n* = 4. (b) Total glutathione in mice livers. Data are expressed as the mean ± SEM. Number of mice: WT: *n* = 6, Sod1^−/−^: *n* = 5, Prdx4^−/y^: *n* = 4, and DKO: *n* = 5. ^∗^ *P* < 0.05 versus WT mice. ^‡^ *P* < 0.05 versus Prdx4^−/y^ mice.

We next examined the oxidative status of the liver by determining the levels of hyperoxidized Prdx (Prdx-SO_2/3_), an oxidative stress marker protein produced by hyperoxidation of the cysteine-sulfhydryl group by hydrogen peroxide [32], as well as Prdx1 to Prdx4 proteins. Prdx1 and Prdx2 proteins are similar in sizes (around 22 kDa) and hence cannot be separated by SDS-PAGE. Thus, the hyperoxidized forms of these isoforms also showed the same electrophoretic mobility and are therefore labeled as Prdx1/2-SO_2/3_ here. Likewise, a hyperoxidized form with a molecular size of 24 kDa, corresponding to a mitochondrial isoform of Prdx3, is labeled Prdx3-SO_2/3_ here. As we reported previously [33], the levels of Prdx1/2-SO_2/3_ were quite low in the WT livers whereas the levels were markedly elevated in the livers of Sod1^−/−^ mice (Figure 5(a)). It is noteworthy that Prdx3 was hyperoxidized to a considerable extent in the livers of Prdx4^−/y^ mice and also in the DKO mice. These results indicate that the Prdx4 and Sod1 deficiency independently disturbed the Prdx redox system.

The elevated levels of hyperoxidized Prdx suggest the overproduction of hydrogen peroxide in the mice that lacked SOD1 and/or Prdx4. Because glutathione is abundant and synthesized in hepatocytes and plays a role in antioxidation via glutathione peroxidases [34], we next examined the issue of whether oxidative stress affected the redox status of DKO mice by measuring the total glutathione level in the livers (Figure 5(b)). Unexpectedly, total glutathione levels were somewhat elevated in Sod1^−/−^ mouse livers and markedly elevated in DKO mouse livers compared to WT or Prdx4^−/y^ mouse livers. Because glutathione synthesis is activated in response to and to combat oxidative stress, the elevation in the glutathione levels suggests that the DKO mouse livers were under continuous oxidative stress. Thus, the elevation in glutathione levels in the DKO mice can be considered to be a compensatory response to oxidative stress. Collectively, these results suggest that the DKO mice suffered the most strongly from oxidative stress due to the double deprivation of the Prdx4 or Sod1 genes.

## 4. Discussion

The findings reported here indicate that, although the deletion of the Prdx4 gene alone had little effect on liver physiology, the deletion of Sod1 together with Prdx4 resulted in an enhanced level of aggravated liver damage compared to Sod1^−/−^ mice (Figures 1 and 2), which reportedly develop liver steatosis [9]. We also showed that an increase in lipid droplet accumulation is spontaneously accelerated in the liver tissue of DKO mice at an early stage of life, due to an impaired triglyceride-rich lipoprotein secretion (Figures 3 and 4), which was associated with heightened oxidative stress in the livers caused by the combined deprivation of SOD1 and Prdx4 (Figure 5). Accelerated lipid droplet formation was consistently observed in the DKO primary hepatocytes, even compared to Sod1^−/−^ hepatocytes (Figure 4).

We found that plasma levels of ALT and AST were increased in the DKO mice (Figures 1 and 1). While ALT is considered to be a specific marker for liver damage, AST may have originated in other tissues, such as skeletal muscle and the kidney. In fact, it has been suggested that age-dependent skeletal muscle atrophy is caused via elevated ROS by the ablation of Sod1 [27], accompanied by the proliferation and differentiation of satellite cells through growth factors in Sod1^−/−^ mice [35]. Because the ablation of SOD1 alone induces sarcopenia but not prominent liver damage at younger ages compared to WT mice [27], the simultaneous absence of Prdx4 would have increased the sensitivity of muscle tissue to ROS and consequently triggered muscular damage earlier than that in single-knockout mice. The kidney is also a potential organ source for plasma AST because ischemia/reperfusion-induced acute renal failure [36] and hypertension in hydronephrosis [37] are aggravated in Sod1^−/−^ mice. In this study, a trend toward an increase in the plasma levels of BUN in Sod1^−/−^ mice and DKO mice was observed, but the values were within the normal range (Supplementary Figure 1). In addition, because the expression of Prdx4 was lower in muscle and renal tissues compared to the liver [38, 39], the ablation of Prdx4 would be expected to influence the liver to a greater extent than these tissues. Thus, it can be reasonably concluded that the origin of the elevated plasma AST levels was from damaged livers and, together with elevated plasma ALT levels, indicates aggravation in the extent of liver damage in DKO mice compared to Sod1^−/−^ mice.

As of this writing, Prdx4^−/y^ mice have shown no signs of tissue damage or abnormalities, except for testicular atrophy [17], and hence liver damage, if any, is latent in them. In this study, we found, for the first time, that the levels of hyperoxidized mitochondrial Prdx3 were markedly elevated in the livers of Prdx4^−/y^ mice (Figure 5(a)). These results were somewhat unexpected because Prdx4 is reportedly present either in the ER lumen [16] or extracellularly in culture media or plasma [14, 15]. The oxidative damage in mitochondrial Prdx3 appeared to support a functional connection between the ER and the mitochondrion where redox reactions play pivotal roles. ER and mitochondria are not directly linked but, rather, are interconnected both physically and functionally via a specific membrane structure, referred to as the mitochondrial-associated ER membrane (MAM) [40]. During ER stress, Ca^2+^ released from the ER is taken up by mitochondria, leading to the release of cytochrome c from the mitochondrial inner membrane. While cardiolipin functions to anchor cytochrome c on the inner mitochondrial membrane, the elevated level of lipid peroxidation of cardiolipin appears to prevent or inhibit this retention and consequently results in the release of cytochrome c to the cytosol [41]. The ablation of Sod1, which is also located in the intermembrane space of mitochondria [4], would result in elevated levels of mitochondrial ROS, thus enhancing oxidative damage. Hence, the simultaneous ablation of Prdx4, which is largely present in the mitochondrial MAM, and Sod1 would enhance oxidative stress and cause an elevation in lipid peroxidation. The released cytochrome c triggers apoptosis by activating apoptosome formation on the one hand, and the loss of cytochrome c inhibits the electron transport chain function, resulting in elevated ROS production, on the other hand [21]. In addition, the failure of disulfide bond formation due to the ablation of the sulfoxidase reaction catalyzed by Prdx4 in the ER would result in the accumulation of misfolded proteins, which would lead to ER stress and the triggering of apoptotic cell death in severe cases [30]. Moreover, even when ER stress is modest, it potentially causes a variety of cellular dysfunctions; for example, a decline in ATP content stimulates mitochondrial respiration and oxygen consumption, which would result in elevated levels of ROS and hence lead to a vicious circle [21]. Meanwhile, hepatic glutathione levels were not altered in the Prdx4^−/y^ mice compared to WT mice (Figure 5(b)), suggesting that the hyperoxidation of Prdx3 alone was in an allowable range and, hence, did not activate the emergency redox system. Prdx2 levels were decreased in livers of each KO mouse compared to WT mouse, and considerable variation was found among individual mice. In fact, we previously reported that hyperoxidized Prdx2 is degraded by the proteasomal system in red blood cells [33]. Hence, the decrease in the DKO mouse hepatocytes appears to reflect accelerated degradation of hyperoxidized Prdx2.

What then is the mechanism responsible for the increased susceptibility of the DKO mice to liver steatosis? As mentioned above, our results indicate that DKO mice suffered from both oxidative stress and ER stress. Oxidative stress induces ER stress and vice versa [21]. Both the correct folding of the apoB protein in the ER and the secretion of the TG-rich lipoprotein VLDL from the liver are inhibited as a result of both ER stress and ROS [21]. Aberrant oxidation due to elevated ROS levels likely causes the misfolding of apoB and disturbs the assembly of VLDL and their secretion from liver, which would lead to the accelerated degradation of misfolded apoB in Sod1^−/−^ mice [9]. ER stress also stimulates lipogenesis by activating transcriptional factors responsible for fatty acid and cholesterol syntheses [42]. This is caused by the activation of the sterol regulatory element-binding proteins (SREBPs), which regulate the expression of genes that are involved in steroidogenesis and lipogenesis [43]. Upon ER stress as well as a sterol insufficiency, SREBPs, which are membrane proteins localized on the ER membrane, are moved to the Golgi membrane, proteolytically activated by the site-1 protease (S1P) and the site-2 protease (S2P) there, and recruited to the nucleus to induce the expression of lipogenic genes [43]. Thus, it is likely that both prolonged oxidative stress and ER stress are synergistically involved in stimulated lipogenesis but the inability to secrete lipoproteins from the liver, thereby resulting in the development of liver steatosis much more severe in the DKO mice than in the Sod1^−/−^ mice. Although we detected liver damage in DKO mice at a relatively young stage, no evidence for the accumulation of type I collagen was found in the aged mice as is typically observed in NASH (data not shown). This would be caused by severe ER stress, which suppresses the excretion of collagen as occurs in mice with multiple deficiencies of Prdx4 and Ero1 [44].

## 5. Conclusion

Using DKO mice in which both Sod1 and Prdx4 were deleted, we show that oxidative stress in association with ER stress leads to the development of a very severe hepatic disorder. It thus appears that these DKO mice have the potential for use as an animal model for unraveling the molecular mechanism responsible for hepatic disorder development from viewpoint of oxidative stress and ER stress and show promise for use in evaluating effective medicines and treatments.

## Abbreviations

NAFLD: Nonalcoholic fatty liver disease
NASH: Nonalcoholic steatohepatitis
ER: Endoplasmic reticulum
ROS: Reactive oxygen species
Sod: Superoxide dismutase
Prdx: Peroxiredoxin
ALT: Alanine aminotransferase
AST: Aspartate transaminase
WT: Wild type
TG: Triglyceride
Prdx-SO_2/3_: Hyperoxidized peroxiredoxins
H&E: Hematoxylin and eosin
CHOP: Endoplasmic reticulum stress-C/EBP homologous protein
VLDL: Very low density lipoproteins
apoB: Apolipoprotein B.

## Supplementary Materials

Supplementary Materials Supplementary Figure 1: plasma levels of BUN in mice. Plasma levels of BUN in mice with the indicated genotypes. Data are expressed as the mean ± SEM. Number of mice: WT: *n* = 5, Sod1^−/−^: *n* = 7, Prdx4^−/y^: *n* = 10, and DKO: *n* = 6. Supplementary Figure 2: levels of proteins involved in ER stress response in primary cultured hepatocytes. Proteins extracted from WT and Prdx4^−/y^ hepatocytes at 24, 48, and 72 h after isolation were subjected to Western blotting using antibodies against IRE1*α*, Bip, PDI, CHOP, Prdx4, and *β*-actin.
