# Supplementary material for: Double Knockout of Peroxiredoxin 4 (Prdx4) and Superoxide Dismutase 1 (Sod1) in Mice Results in Severe Liver Failure
Source: Oxid Med Cell Longev. 2018 Jun 27;2018:2812904. doi: 10.1155/2018/2812904 (PMC6040270; doi:10.1155/2018/2812904)
Supplement: Supplementary Materials — Supplementary Figure 1: plasma levels of BUN in mice. Plasma levels of BUN in mice with the indicated genotypes. Data are expressed as the mean ± SEM. Number of mice: WT: n = 5, Sod1−/−: n = 7, Prdx4−/y: n = 10, and DKO: n = 6. Supplementary Figure 2: levels of proteins involved in ER stress response in primary cultured hepatocytes. Proteins extracted from WT and Prdx4−/y hepatocytes at 24, 48, and 72 h after isolation were subjected to Western blotting using antibodies against IRE1α, Bip, PDI, CHOP, Prdx4, and β-actin. [file 2812904.f1.pptx]

## Slide 1
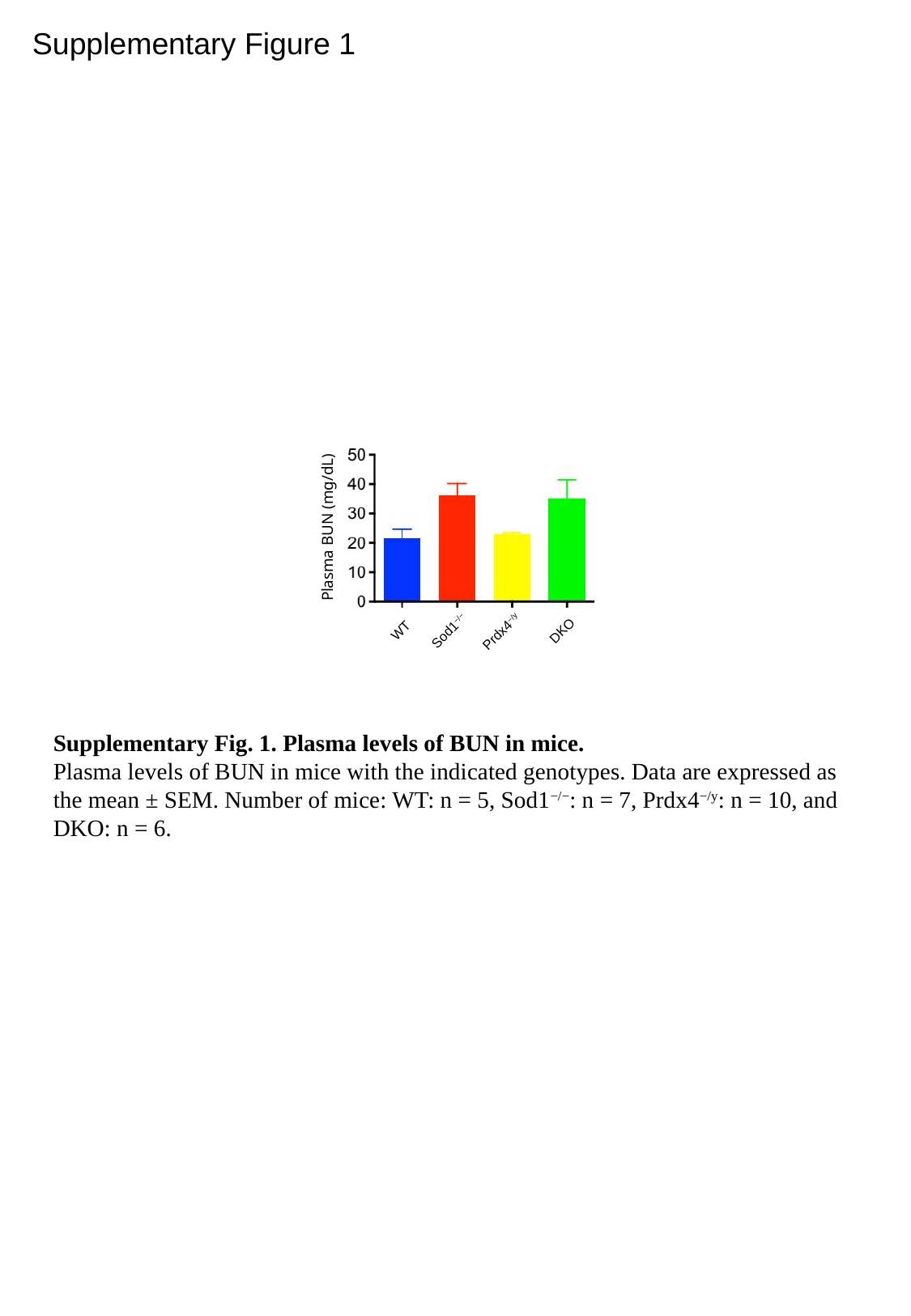

Supplementary Figure 1
Plasma BUN (mg/dL)
WT
Sod1−/−
Prdx4−/y
DKO
Supplementary Fig. 1. Plasma levels of BUN in mice.
Plasma levels of BUN in mice with the indicated genotypes. Data are expressed as the mean ± SEM. Number of mice: WT: n = 5, Sod1−/−: n = 7, Prdx4−/y: n = 10, and DKO: n = 6.

## Slide 2
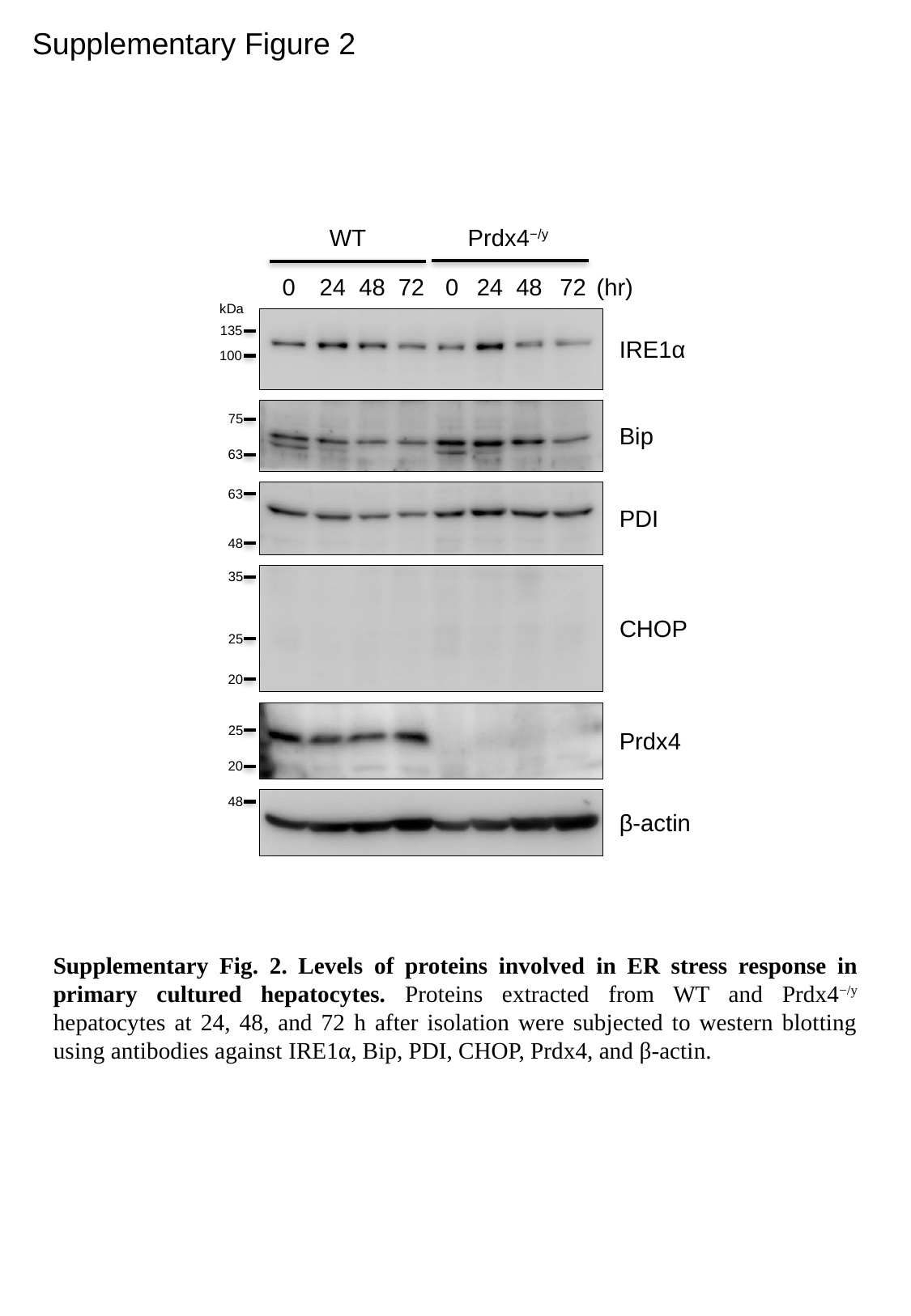

Supplementary Figure 2
WT
Prdx4−/y
0
24
48
72
0
24
48
72
(hr)
kDa
135
IRE1α
100
75
Bip
63
63
PDI
48
35
CHOP
25
20
25
Prdx4
20
48
β-actin
Supplementary Fig. 2. Levels of proteins involved in ER stress response in primary cultured hepatocytes. Proteins extracted from WT and Prdx4−/y hepatocytes at 24, 48, and 72 h after isolation were subjected to western blotting using antibodies against IRE1α, Bip, PDI, CHOP, Prdx4, and β-actin.
